# Long noncoding RNAs associated with nonalcoholic fatty liver disease in a high cholesterol diet adult zebrafish model

**DOI:** 10.1038/s41598-021-02455-0

**Published:** 2021-11-26

**Authors:** Hyo Jung An, Yoon Jung Lee, Chong Pyo Choe, Hyun-Kyung Cho, Dae Hyun Song

**Affiliations:** 1 Department of Pathology, Gyeongsang National University Changwon Hospital, Changwon, Republic of Korea; 2 Department of Pathology, Gyeongsang National University School of Medicine, 15 Jinju-daero 816 Beon-gil, Jinju, 660-751 Korea; 3 Department of Pathology, College of Medicine, Institute of Health Science, Gyeongsang National University, Jinju, Republic of Korea; 4 Division of Life Science, Gyeongsang National University, Jinju, 52828 Republic of Korea; 5 Division of Applied Life Science, Plant Molecular Biology and Biotechnology Research Center, Gyeongsang National University, Jinju, Republic of Korea; 6 Department of Ophthalmology, Gyeongsang National University Changwon Hospital, Gyeongsang National University, School of Medicine, Changwon, Republic of Korea; 7 Institute of Health Sciences, School of Medicine, Gyeongsang National University, Jinju, Republic of Korea

**Keywords:** Biological techniques, Computational biology and bioinformatics, Genetics, Molecular biology, Zoology, Health care, Medical research, Molecular medicine, Pathogenesis

## Abstract

The mechanism of nonalcoholic fatty liver disease (NAFLD) has not been completely revealed. In this study, we investigated the association of liver histological changes and long noncoding RNAs (lncRNAs) in the NAFLD zebrafish model. Forty zebrafish were fed a high-cholesterol diet (1.5 g per day) for 8 weeks. We measured fatty liver changes in the zebrafish liver using oil red O staining and divided them into two groups based on high and low scores. We pooled each group of zebrafish livers and identified lncRNAs, miRNAs, and mRNAs using Next-generation sequencing. Human homologs of lncRNAs were identified using ZFLNC, Ensembl, and NONCODE. We found several significant genes, including 32 lncRNAs, 5 miRNA genes, and 8 protein-coding genes, that were associated with liver metabolism and NAFLD-related functions in zebrafish. In particular, eight conserved human homologs of lncRNAs were found. We discovered the human homologs of eight lncRNA candidates from fatty liver zebrafish for the first time. The spectrum of biological mechanisms by which lncRNAs mediate their functional roles in NAFLD in a high cholesterol diet adult zebrafish model remains to be uncovered.

## Introduction

Obesity is a serious and important condition associated with various metabolic diseases. Nonalcoholic fatty liver disease (NAFLD) is one of the fatal diseases that is mainly caused by obesity and is known to have a prevalence of 20% to 30% among the general population around the world^1^. NAFLD is a spectrum of conditions ranging from steatosis to nonalcoholic steatohepatitis (inflammation), liver cirrhosis (fibrosis), and finally hepatocellular carcinoma^1,2^. However, the mechanism of NAFLD has not been completely revealed due to the complexity of the outbreak and asymptomatic features. The progression of NAFLD from steatosis to fibrosis is thought to be a so-called multihit mechanism, with both environmental and genetic factors known to be involved^3,4^. Among them, lncRNA (long non-coding RNA) is emerging as an essential genetic factor that operates and contributes to the process of NAFLD fibrosis^5^. More interestingly, certain miRNAs and lncRNAs contribute to histological changes in NAFLD by modulating homeostasis in body fat^6^. LncRNAs are defined as nonprotein-coding transcripts that are more than 200 nucleotides in length. Until recently, only a small number of lncRNAs have been functionally characterized, and the molecular mechanisms of lncRNAs in human diseases have remained unclear^7^. However, since approximately 2014, they have been shown to be related to important biological functions, such as cancer cell proliferation, cell differentiation, and cell development and aging^7–10^. Briefly, the functions of lncRNAs can be summarized as (1) epigenetic changes, (2) transcriptional regulation, and (3) posttranscriptional regulation. In addition, a subset of lncRNAs could interact with smaller RNAs, including miRNAs, to modulate their regulatory effects^11,12^. Previously, authors have validated the regulatory interactions between a conserved pair including a lncRNA and a miRNA in zebrafish^13^.

**Figure 1:**
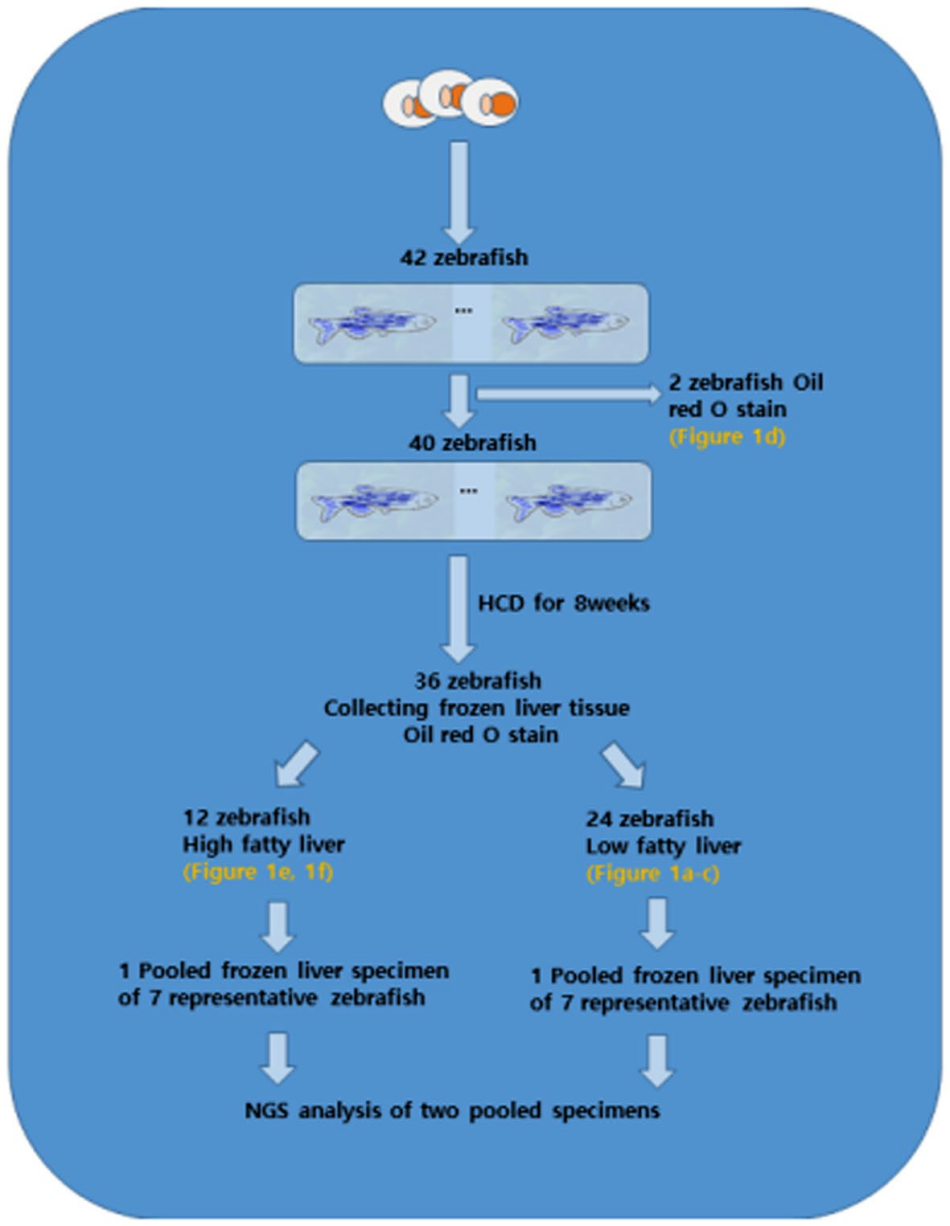
Workflow diagram of the feeding and tissue processing of zebrafish.

lncRNAs are highly conserved due to their short lengths to preserve functional or secondary structures^13^. Although very few lncRNAs exhibit sequence conservation across species^14,15^, since lncRNAs contribute more to cell-line specificity than protein-coding genes proven by Djebali et al.^16^, meaningful research on lncRNAs associated with NAFLD is possible if one can find lncRNAs in certain animals that are homologous in humans. In this work, to identify lncRNAs in the NAFLD zebrafish model, we performed high-cholesterol diet feeding, measured and grouped NAFLD in the liver of zebrafish, and performed next-generation sequencing in the zebrafish liver (Fig. 1). Using bioinformatics approaches, we found eight human homologs of lncRNA candidates that might be associated with liver metabolism and NAFLD.

## Results

### Nonalcoholic fatty liver disease in the adult zebrafish model fed a high-cholesterol diet

**Figure 2:**
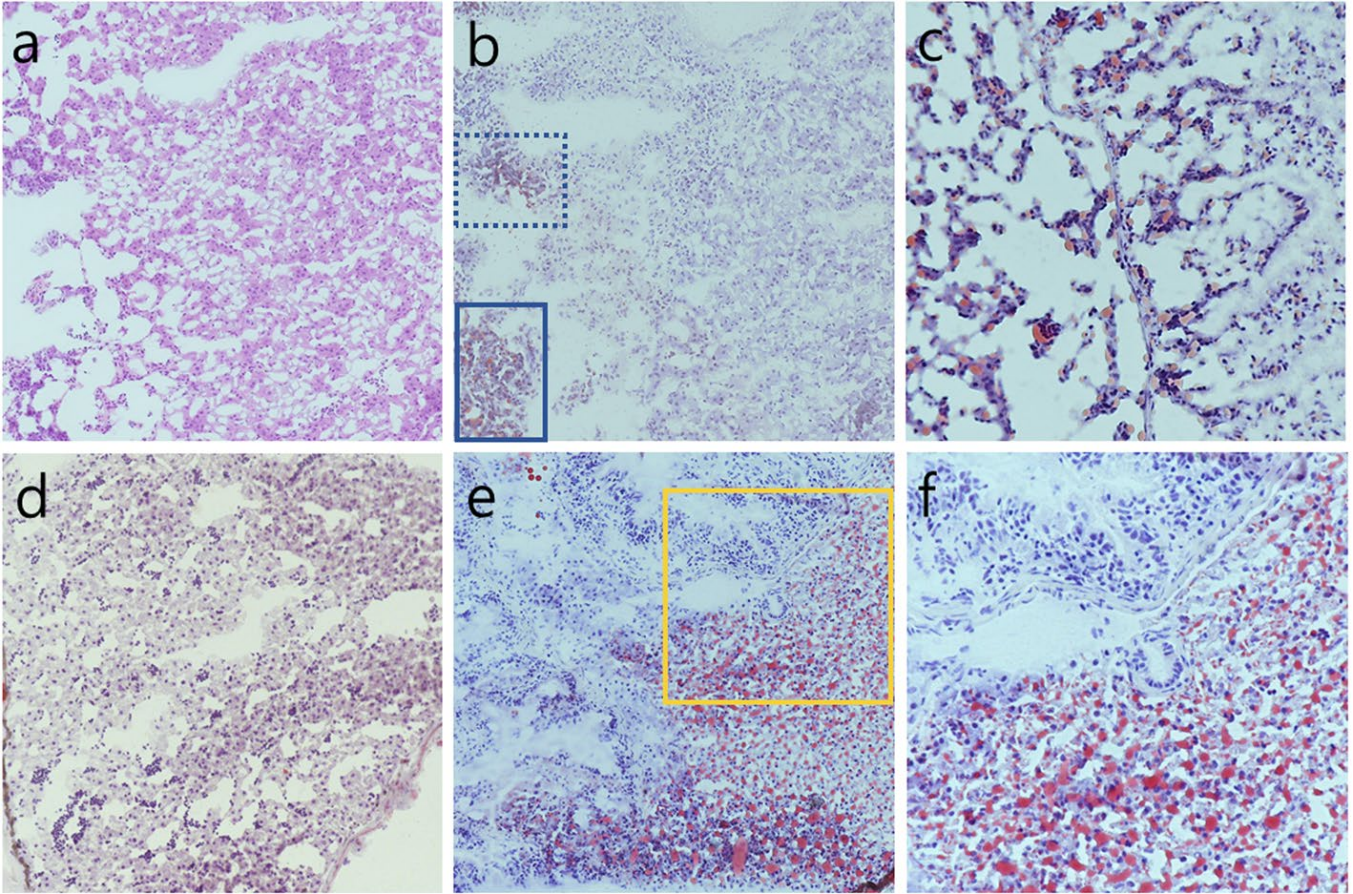
A representative frozen section (× 200, hematoxylin and eosin) **(a)** of a liver was obtained from zebrafish No. 5 (low fatty liver group) and stained with oil red O. In the same area of Fig. 1a, a 10% fatty change was observed with oil red O positive stain (box) (× 200) **(b)**. Higher magnification of the oil red O positive area (lower box marked with solid line in Fig. 1b) (× 400) **(c)**. A representative section of oil red O stain from the zebrafish before feeding a high cholesterol diet (× 200) **(d)** (see the workflow diagram of Fig. 1). The high fatty liver group contains zebrafish that have a proportion score of no less than 50 (× 200) **(e)**. At higher magnification (yellow box in Fig. 1e), both macrovesicular and microvesicular fatty changes were detected at high levels in the high fatty liver group compared to the negative internal control of the glandular epithelial cells in the stomach (left upper side) (× 400) **(f)**.

The fatty liver change scores of 36 adult zebrafish after 8 weeks of a high-cholesterol diet are summarized in Table 1. For example, 6 frozen sections (Fig. 2a) of a liver were obtained from zebrafish No. 5 and stained with oil red O. If the 10% fatty change in oil red O was the highest among the six slides, it became the representative score of zebrafish No. 5 (Fig. 2b,c). A representative section of oil red O stain from the zebrafish before feeding high cholesterol diet showed negative expression (Fig. 2d) (see the workflow diagram of Fig. 1). All zebrafish sections after the high cholesterol diet were stained with oil red O, divided into two groups: the low and the high fatty liver groups. The high group contained zebrafish that have proportion scores of no less than 50 (Fig. 2e). At higher magnification, in the representative higher score group, both macrovesicular and microvesicular fatty changes were severely detected compared to the negative internal control (Fig. 2f).

**Table 1:** Fatty change scores of 36 adult zebrafish after 8 weeks of high cholesterol diet.

No. of Z	1	2	3	4	5	6	7	8	9	10	11	12	13	14	15	16	17	18	19	20	21	22	23	24	25	26	27	28	29	30	31	32	33	34	35	36
Fatty^a^ liver (%)	10	60	60	80	10	30	40	50	30	20	10	20	10	10	70	30	15	20	20	5	60	80	10	20	70	40	10	20	5	40	60	80	10	90	20	70
Group^b^	L	H	H	H	L	L	L	H	L	L	L	L	L	L	H	L	L	L	L	L	H	H	L	L	H	L	L	L	L	L	H	H	L	H	L	H
NGS group^c^	L			H	L						L		L	L	H					L		H			H				L			H		H		H

^a^The proportion of fatty change in liver was measured by two pathologists. More than 6 sections of a liver of a zebrafish were obtained and stained by Oil red O. The highest proportion value among 6 sections is representative score of fatty liver at each zebrafish. For example, 6 frozen sections of a liver were obtained from No.1 zebrafish, stained by Oil red O. 10% of fatty change in oil red O is the highest and representative score among 6 liver sections of No.1 zebrafish.

^b^All zebrafishes were divided as two groups. H (high) group contains zebrafishes have not less than 50 proportion score.

^c^Seven zebrafishes which have the highest proportion score of fatty liver were pooled as high group in RNA sequencing analysis.

*Z* zebrafish, *No.* number, *H* high, *L* low, *NGS* next-generation sequencing analysis.

### Long noncoding RNAs associated with nonalcoholic fatty liver disease in zebrafish

**Figure 3:**
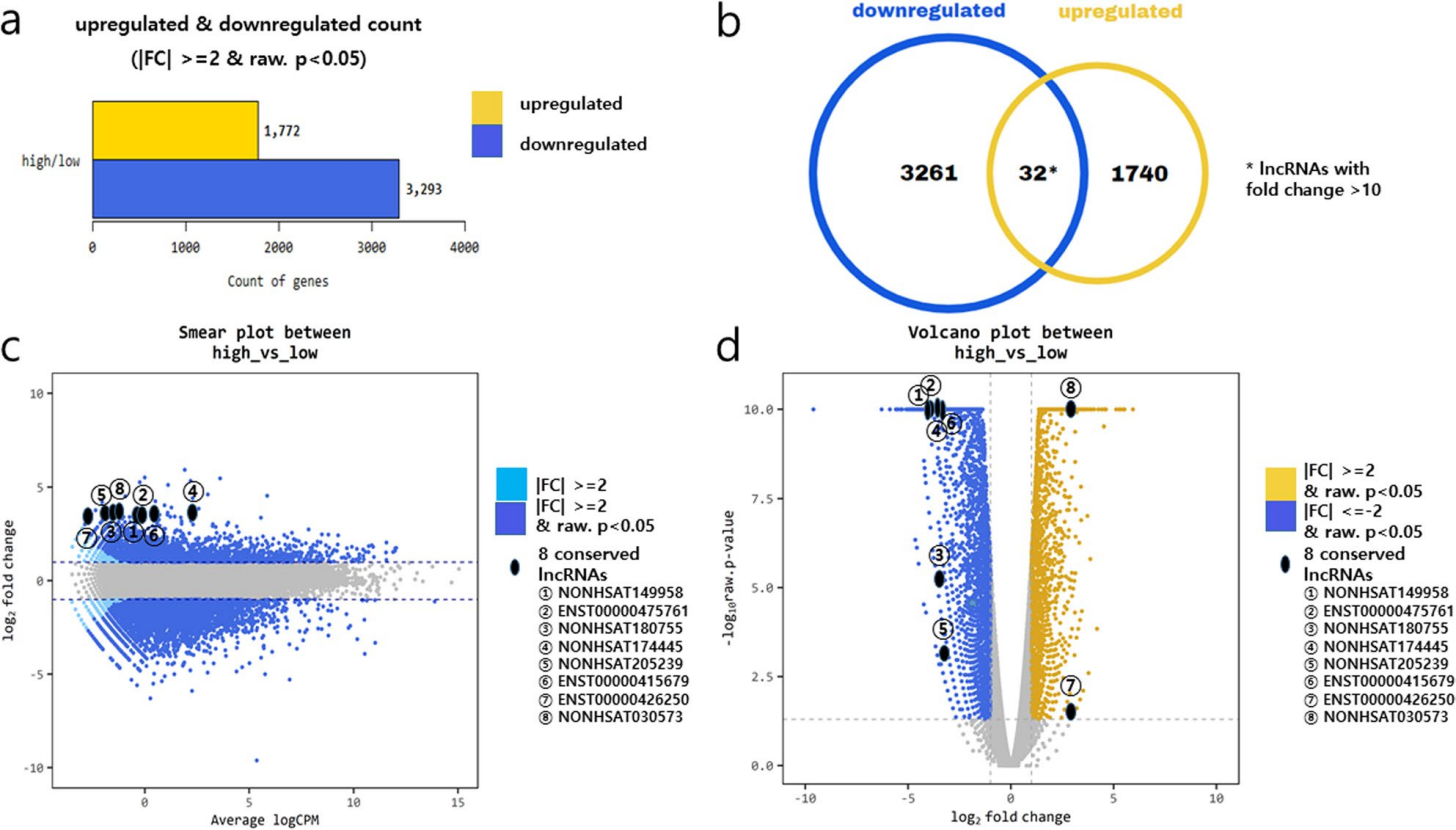
A total of 1772 genes were upregulated and 3293 genes were downregulated with statistical significance **(a,b).** In the Venn diagram, upregulated or downregulated lncRNAs with more than tenfold changes are featured **(b)**. The smear plot **(c)** and volcano plot **(d)** between the high and low fatty change groups are shown. Eight conserved lncRNAs are placed and featured in each plot **(c,d)**.

After the library preparation and validation quality check, the high fatty change group and low fatty change group were included in the final RNA sequencing. Through a next-generation sequencing approach, differentially expressed gene (DEG) analyses between high and low fatty change group using edgeR was conducted and 5065 selected genes satisfying the |fold change| ≥ 2 and p-value < 0.05 condition was extracted. The heatmap using the Z-score of the log 2 based Top 8 differentially expressed protein-coding genes with normalized value by Morpheus Software (https://software.broadinstitute.org/morpheus/) is shown in supplementary data 1. The Gene ontology analyses for a significant DEG list associated with NAFLD in zebrafish were conducted using gProfiler (https://biit.cs.ut.ee/gprofiler/orth). Gene set enrichment analysis for each biological process (BP), molecular function (MF), and cellular component (CC), which are functional classifications of gene ontology are shown in supplementary data 2. Among 5065 genes, 1772 genes were upregulated and 3293 genes were downregulated with statistical significance (Fig. 3a,b). The smear plot and volcano plot between the high- and low- fatty change groups are featured in Fig. 3c,d. We selected 548 lncRNAs that had a greater than twofold change, either downregulated or upregulated, than the control group with statistical significance with a p-value of less than 0.05. Then, we chose 28 downregulated and 8 upregulated lncRNAs that had greater than tenfold changes than the control group with p-values of less than 0.05 (Fig. 3b). The identified 28 lncRNAs are listed in Table 2.

**Table 2:** Next-generation sequencing analysis; 8 candidates of human homologs by ZFLNC.

Gene ID (NCBI)	Transcript_ID (NCBI)	Transcript_ID (ZFLNC)	Gene ID (ZFLNC)	Score by ZFLNC BLASTN 2.7.1 +	Identical % by ZFLNC BLASTN 2.7.1 +	Human homology by ZFLNC	H/L fold change	H/L p
110438940	XR_002457343	ZFLNCT11405		946	87.6%		-45.17	4.9E-20
110439701	XR_002458698	ZFLNCT00156		2562	100%		-27.20	1.9E-13
101882800	XR_224743	ZFLNCT17597		178	82.6%		-18.93	3E-05
103911713	XR_662734	ZFLNCT09503		1280	99.8%		-18.39	4.4E-09
108191363	XR_001800351	ZFLNCT11788		1790	93.8%		-16.56	3E-08
**100333843**	**XR_001797020**	**ZFLNCT18125**	**ZFLNCG11696**	36,864	100%	**NONHSAT149958**	-15.84	1.4E-12
100150353	XR_002456541	ZFLNCT09503		960	98.4%		-15.82	7.8E-08
101886926	XR_001796892	ZFLNCT17255		13,492	99.8%		-12.97	7E-25
**108190102**	**XR_002458921**	**ZFLNCT14973**	**ZFLNCG09725**	8454	98.5%	**ENST00000475761**	-12.93	6.4E-14
**110439933**	**XR_002459206**	**ZFLNCT11394**	**ZFLNCG07435**	1586	92.9%	**NONHSAT180755**	-12.89	2.4E-06
**103910640**	**XR_002458426**	**ZFLNCT04118**	**ZFNLCG02650**	6080	100%	**NONHSAT174445**	-12.66	2.8E-34
101883787	XR_002458833	ZFLNCT13363		3644	99.4%		-12.65	0.00045
101885609	XR_659302	ZFLNCT02477		212	77.9%		-12.65	0.00045
110438361	XR_002456650	ZFLNCT20450		1318	99.8%		-12.53	3.7E-15
108190381	XR_001799550	ZFLNCT05947		564	99%		-12.16	6.4E-06
**108190704**	**XR_001799947**	**ZFLNCT10393**	**ZFLNCG06796**	3090	95.5%	**NONHSAT205239**	-11.26	0.00141
108180259	XR_001796785	ZFLNCT17078		544	100%		-11.05	8.7E-22
108179803	XR_001796363	ZFLNCT06058		1820	99.5%		-10.98	2.3E-07
101882203	XR_002456302	ZFLNCT11783		3476	92.4%		-10.91	1.2E-11
103909331	XR_659184	ZFLNCT17178		41,024	100%		-10.73	3.7E-07
**101883877**	**XR_001796542**	**ZFLNCT15818**	**ZFLNCG10246**	65,078	100%	**ENST00000415679**	-10.71	7.3E-15
101882015	XR_224239	ZFLNCT08748		2738	94.2%		-10.69	4.7E-05
110440015	XR_002459399	ZFLNCT20292		1874	91.7%		-10.49	5.9E-07
103908766	XR_001801033	ZFLNCT04771		9526	94.5%		-10.32	7.7E-05
**103910542**	**XR_660943**	**ZFLNCT03884**	**ZFLNCG02476**	3334	95.1%	**ENST00000426250**	11.45	0.00788
101886765	XR_223052	ZFLNCT20133		6198	100%		11.52	2E-18
100536168	XR_002458879	ZFLNCT05009		11,036	100%		12.42	1.1E-22
**110440075**	**XR_002459626**	**ZFLNCT04874**	**ZFLNCG03169**	884	88.1%	**NONHSAT030573**	13.35	4.2E-11
103910598	XR_002458457	ZFLNCT14983		356	97.9%		13.72	0.0025
110439000	XR_002457406	ZFLNCT10919		54	76.3%		18.25	0.00014
108180020	XR_001796501	ZFLNCT02106		266	85.9%		21.37	2.3E-41
110438681	XR_002457081	.Not identified					45.95	8.3E-19

*H* high, *L* low, *p* p value, *E−20*  × 10^–20^.

### Identification of human homologs of eight long noncoding RNAs of zebrafish

**Figure 4:**
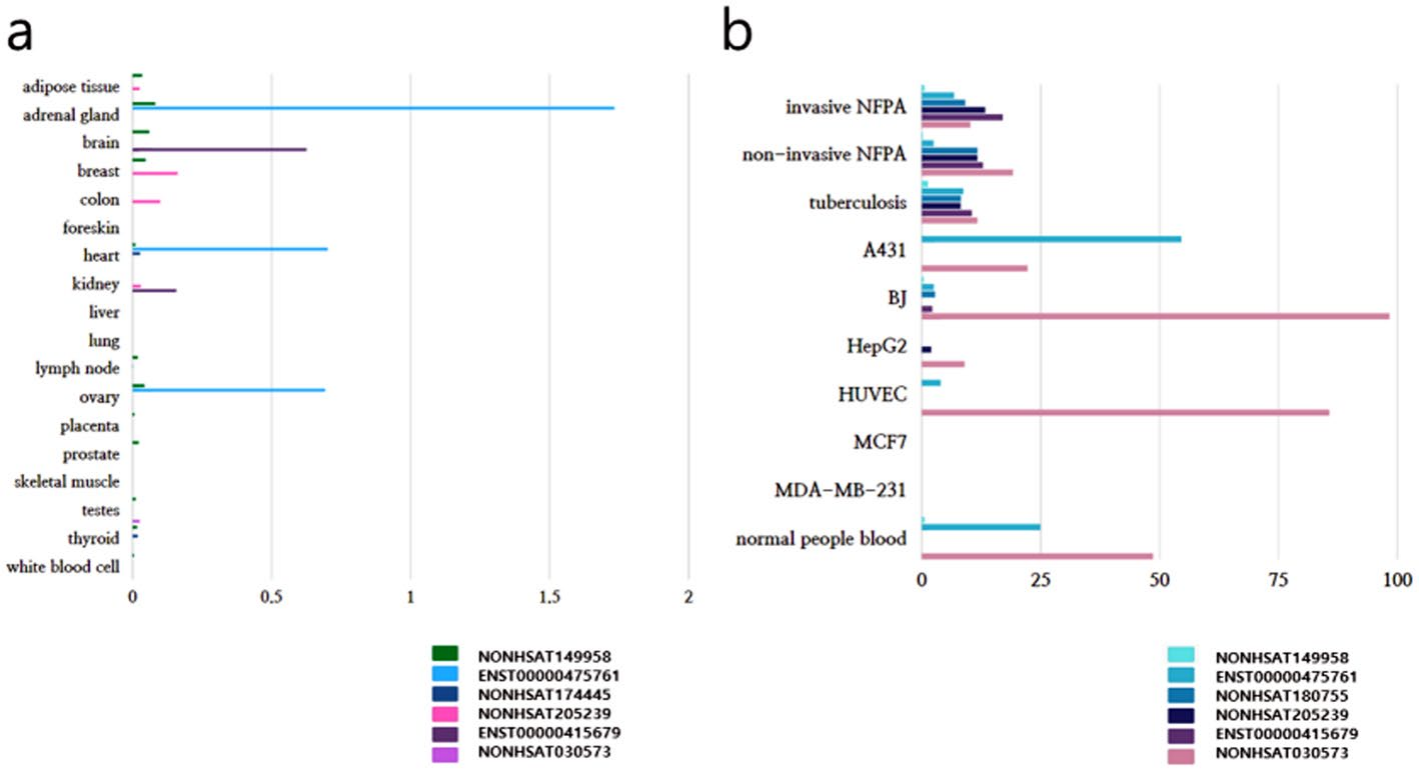
The human tissue expression profiles represented by FPKM/TPM **(a)** and exosome expression profiles represented by FPKM **(b)** were evaluated on 8 conserved lncRNAs. invasive NFPAs, invasive nonfunctional pituitary adenomas exosomes; non-invasive NFPAs, non-invasive nonfunctional pituitary adenoma exosomes; tuberculosis, active tuberculosis patient serum exosomes; A431, squamous cell carcinoma cell line exosomes; BJ, foreskin fibroblast cell line exosomes; HepG2, hepatocellular carcinoma cell lines; MCF7, human breast cancer cell line exosomes; MDA-MB-23a, human breast cancer cell line exosomes; normal people blood, normal people blood exosomes (http://www.noncode.org/).

To obtain the human homologs of each lncRNA of the NAFLD zebrafish, we performed ZFLNC BLAST and NCBI FASTA. First, in the NCBI section, we copied the sequence of the lncRNA and put it in the BLAST section of ZFLNC. The genes with the highest score were designated human homologs of the specific lncRNAs. Eight (ZFLNCT18125, ZFLNCT14973, ZFLNCT11394, ZFLNCT04118, ZFLNCT10393, ZFLNCT15818, ZFLNCT03884, and ZFLNCT04874) out of 28 lncRNAs were confirmed to be conserved, as evaluated by ZFLNC BLASTN.2.7.1 + (Table 2). The human homologs of eight lncRNAs according to ZFLNC are NONHSAT149958, ENST00000475761, NONHSAT180755, NONHSAT174445, NONHSAT205239, ENST00000415679, ENST00000426250, and NONHSAT030573 (Table 2). We searched these human loci in the Ensembl database (http://www.ensembl.org) and the NONCODE database (http://www.noncode.org) to obtain general information, sequences, expression profiles in human tissues, and exosome expression profiles. We put the FASTA link for each conserved lncRNAs in supplementary data 3. Since these genes are specific lncRNA candidates that were identified for the first time, there is limited information, especially regarding their structures. However, among the eight conserved lncRNAs, six lncRNAs were downregulated and two lncRNAs were upregulated in the zebrafish liver with high fatty changes compared to that of low fatty changes, as demonstrated by Next-generation sequencing and ZFLNC. The human tissue expression profiles represented by FPKM/TPM (Fig. 4a) and exosome expression profiles represented by FPKM (Fig. 4b) were evaluated for each conserved lncRNA.

### Identification of miRNA genes and protein-coding genes in zebrafish fed a high-cholesterol diet using Next-generation sequencing

**Figure 5:**
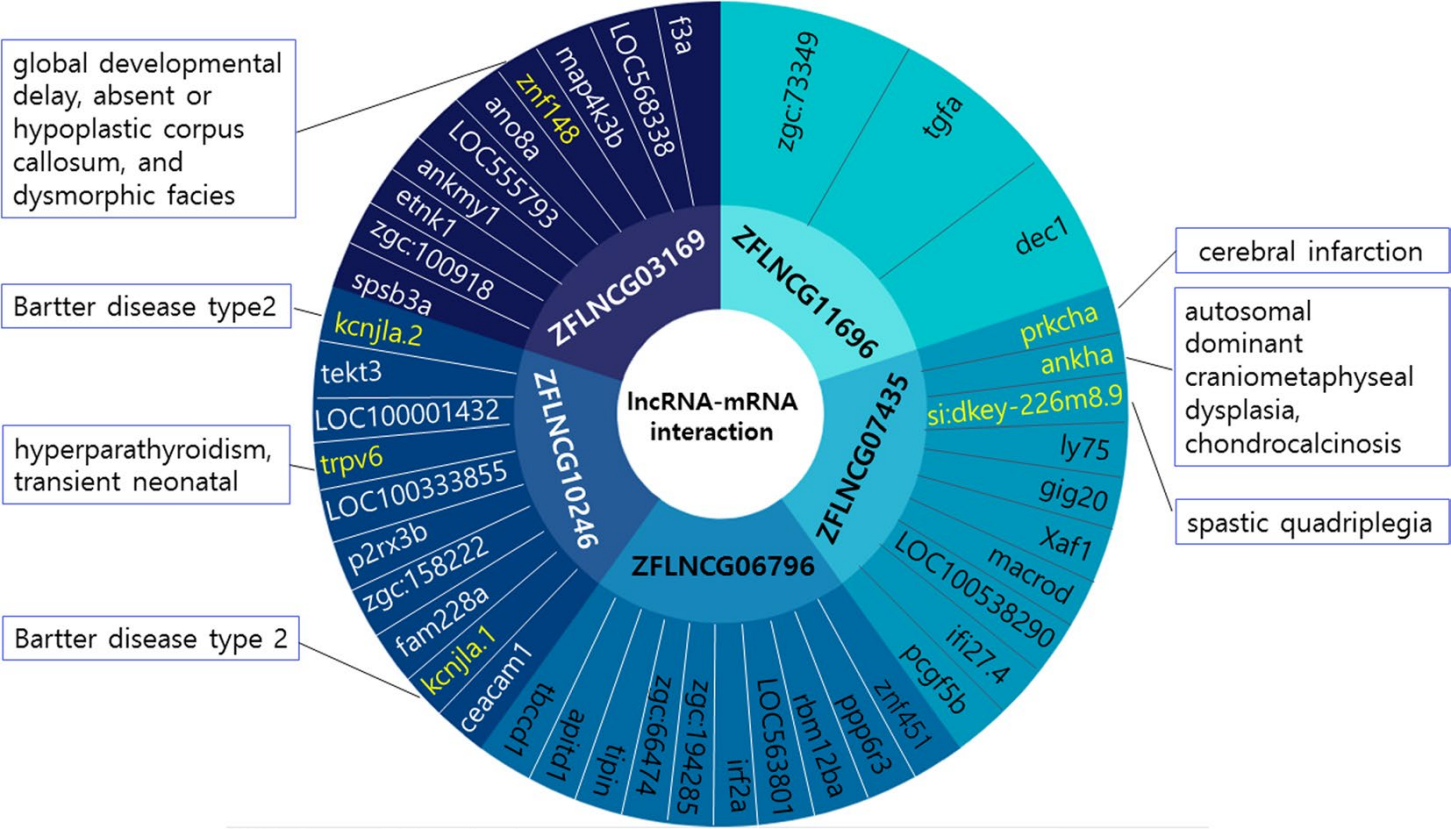
lncRNA–mRNA (protein-coding gene) interaction network for 5 out of 8 conserved lncRNAs in zebrafish. Among the eight candidates, five (ZFLNCG11695, ZFLNCG07435, ZFLNCG06796, ZFLNCG10246, and ZFLNCG03169) of which were identified as having related protein-coding genes of zebrafish, and three (ZFLNCG07435, ZFLNCG10246, and ZFLNCG03169) of which were identified as having genes (prkcha, ankha, si:dkey-226m8.9, kcnjla.2, trpv6, kcnjla.1, and znf148) related to human disorder. Information provided by ZFLNC (http://www.zflnc.org/) and zfin (https://zfin.org/).

Through a next-generation sequencing approach, among the 5065 genes that were associated with NAFLD of zebrafish, we detected 5 miRNA genes and 8 protein coding genes that had greater than a 40-fold change than the lower score group, either downregulated or upregulated. Among them, two miRNA genes and 8 protein-coding genes showed a statistically significant difference from the lower cholesterol group (p-values less than 0.05). However, we could not find the human homologs of each miRNA and protein-coding gene. The identified miRNA and protein-coding gene are listed in Table 3. In contrast, we found that there was a lncRNA-mRNA (protein-coding gene) interaction network among 5 out of 8 conserved lncRNAs in the zebrafish using ZFLNC (http://www.zflnc.org/) (Fig. 5).

**Table 3:** Next-generation sequencing analysis; differentiation of miRNA genes and protein coding genes between the high and low groups.

	Gene ID (NCBI)	Transcript_ID (NCBI)	ZFIN	H/L fold change	H/L p
miRNA genes	100033753	NR_030522	ZDB-GENE-090929-319	−7.07	0.075
	100033671	NR_034203	ZDB-GENE-090929-158	−3.28	0.016
	100033538	NR_029981	ZDB-GENE-081203-2	2.56	0.130
	100310767	NR_030652		9.19	0.075
	100033598	NR_030031	ZDB-GENE-081210-6	10.31	0.047
Protein coding genes	100141330	NM_001114895	ZDB-GENE-080218-6	−782.88	3.9E−126*
	108179280	XM_021466319		−78.23	9.6E−19
	64671	NM_131692	ZDB-GENE-030522-1	−59.28	8.4E−45
	100034392	XM_021468316	ZDB-GENE-060503-24	−48.23	5.4E−12
	557378	XM_021471206	ZDB-GENE-041210-279	−41.34	9.4E−23
	100147849	XM_021477626		40.62	6.5E−45
	108180038	XM_021467837		44.49	1.7E−66
	100331508	XM_009302838		61.28	8.8E−44

*p* p value, *, E−126 × 10^–126^.

## Discussion

Given its clinical importance and enormous socioeconomic impacts, the underlying mechanism, as well as various clinical interventions, have been extensively studied for NAFLD. However, since the pathophysiological mechanism is very complex, drugs that directly act on NAFLD have yet to be developed. Traditional methods of developing NAFLD drugs include in vitro cell line experiments and in vivo mammalian models, such as mice^17^. In the case of mouse models, it takes a long time to observe pathological lesions in the liver through the standard high-fat diet in which mice start to have fatty liver changes from the 8th week to the 12th week and a nonalcoholic steatohepatitis feature at the 12th week^18^. In addition, it is difficult to proceed due to the high cost and low capacity of newly developed drugs. Moreover, in vitro cell line experiments are limited to multiple-stage NAFLD^17^. Therefore, an in vivo model is needed that can proceed with a high-fat diet in a short period of time and can be used to screen the effectiveness of a drug with a small amount of NAFLD medicine. Recently, a high-cholesterol diet-induced larval zebrafish model (Danio rerio) was highlighted as a screening drug for the progression of NAFLD^19^. In addition, the model was used to perform mechanistic research. Therefore, in this work, we wanted to explore the association of fatty liver changes and lncRNAs in the NAFLD zebrafish model. We fed a high-cholesterol diet to wild-type adult zebrafish, measured the occurrence of NAFLD in the liver of zebrafish, and checked the lncRNAs associated with NAFLD outbreaks using Next-generation sequencing in the liver of zebrafish. Then, bioinformatics approaches, including ZFLNC (ZFLNC BLASTN 2.7.1 +), Ensembl, and NONCODE were undertaken as part of this study. We found eight conserved lncRNA candidates that might be associated with liver metabolism and NAFLD-related functions. ZFLNC is a database of vast amounts of information and knowledge about zebrafish lncRNAs. It is a well-annotated online system developed by Xiang Hu et al. in 2018 to provide conserved mammalian counterparts for zebrafish lncRNAs^20^. The principal data resources of zebrafish lncRNAs were Ensembl, NONCODE, NCBI, zflncRNApedia, and other literature. In addition, the authors obtained lncRNA annotations by gathering and comparing each of the expression profiles, coexpression networks, Gene Ontology (GO), Kyoto Encyclopedia of Genes and Genomes (KEGG), Online Mendelian Inheritance in Man (OMIM), and conservation analysis. They developed the database architecture in the Ubuntu Linux server and functionalized each menu, including BLAST, CONSERVATION, Gbrowser, OMIM, and ID conversion^20^. ZFLNC has grown to be one of the most comprehensive platforms covering expression profiles, correlated coding genes, gene ontology, KEGG pathways, conservation, and OMIM and includes the largest number of genes (13,604) and transcripts (21,128) of zebrafish lncRNAs.

From an evolutionary point of view, conserved sequences are similar or identical in RNA, DNA, or proteins across a species, within a genome, or in gene transfer between two organisms. Conservation, in the evolutionary sense, means that a sequence has been genetically preserved due to natural selection. From generation to generation, nucleic acid sequences can be gradually changed due to deletions and random mutations^21,22^. In addition, when chromosomal rearrangement occurs, sequences may be recombined or deleted. Conserved sequences, unlike other sequences, are rarely mutated and preserved within the genome^23^. Either coding or noncoding sequences are conserved. Noncoding genes, which are essential for gene regulation, are conserved in a genome. In lncRNAs, however, sequence conservation generally does not occur as well as in protein-coding genes, and instead, sequences related to secondary structure or function are primarily conserved^24,25^. Nevertheless, the sequence conservation of lncRNAs is meaningful. While the protein-coding gene or amino acid sequence requires a strong functional restriction in the length or number of genes, lncRNAs, due to their relatively short structures of length, are highly conserved, preserving their functional or secondary structure^13^. Although very few lncRNAs exhibit sequence conservation across species, meaningful research on lncRNAs associated with certain diseases is possible if we can identify lncRNAs in certain animals that are homologous in humans. This method has the advantage of allowing experiments to be conducted in animals with a conserved gene that cannot be tested in humans for economic or ethical reasons. Herein, we conducted our study using bioinformatics approaches, including ZFLNC, Ensembl, and NONCODE. We found several significant genes that were associated with liver metabolism and NAFLD-related functions in zebrafish. Specifically, eight conserved lncRNAs were found to have homologs in humans (Table 2).

Eight conserved lncRNAs are either downregulated (6 lncRNAs) or upregulated (2 lncRNAs) in the liver with high fatty changes compared to that in the liver with low fatty changes, as demonstrated by Next-generation sequencing. The 6 downregulated lncRNAs are expected to protect or prevent NAFLD, and the 2 upregulated lncRNAs are expected to cause fatty changes in the liver. The human tissue expression profiles represented by FPKM/TPM based on data from the human BodyMap (Fig. 4a) and exosome expression profiles represented by FPKM based on data from NCBI GEO (Fig. 4b) were evaluated for each conserved lncRNA. A few lncRNAs have human tissue expression profiles in adipose tissue (NONHSAT149958, and NONHSAT205239). It is not clear whether they contribute to the mechanisms affecting fatty changes in the liver with the information described above. In addition, there were two lncRNAs (NONHSAT205239, and NONHASTAT030573) with exosome expression profiles in HepG2 cells (hepatocellular carcinoma cell line exosomes). Since NONHSAT205239 has both adipose tissue expression and an exosome expression profile from the hepatocellular carcinoma cell line, it will be the most powerful candidate for revealing the mechanistic potential for NAFLD. In addition, we found that there was a lncRNA-mRNA (protein-coding gene) interaction network among 5 out of 8 conserved lncRNAs in the zebrafish (Fig. 5). Among the eight candidates, the information provided by ZFLNC (http://www.zflnc.org/) and zfin (https://zfin.org/) was identified, five (ZFLNCG11695, ZFLNCG07435, ZFLNCG06796, ZFLNCG10246, and ZFLNCG03169) of which were identified as having related protein-coding genes of zebrafish, and three (ZFLNCG07435, ZFLNCG10246, and ZFLNCG03169) of which were identified as having genes (prkcha, ankha, si:dkey-226m8.9, kcnjla.2, trpv6, kcnjla.1, and znf148) related to human disorder. Evaluating the regulatory interactions between those genes may help to identify biological mechanisms and treat NAFLD.

To date, the effects and mechanisms of lncRNA-mediated regulation in the pathogenesis of a particular disease have not been properly identified, especially the effects of lncRNAs on NAFLD. Recently, a regulatory interaction between a conserved pair of miRNAs and lncRNAs in zebrafish was discovered^13^. In addition, coding RNA–miRNA–lncRNA network and ceRNA network have been analyzed to discover the effect of tumor microenvironments in 1p/19q codeletion in oligodendrogliomas^26^. In this study, we discovered human homologs of eight lncRNA candidates from the fatty liver of zebrafish for the first time. Although we could not evaluate all of the lncRNAs, up to 547 genes identified by Next-generation sequencing, we identified human homologs of eight out of 32 lncRNAs, between high and low-fatty zebrafish liver groups. Since information on zebrafish lncRNA has not yet been properly organized, there is a limit to building a ceRNA network or finding the regulatory interaction of miRNA in a specific conserved lncRNA. In this study, the zebrafish gene was used to identify conserved lncRNAs for NAFLD mechanical research, and the detailed and practical methods for NAFLD animal model experiments were elucidated. Using a high cholesterol diet adult zebrafish model, we expect to uncover the biological mechanism and treatment by which lncRNA candidates mediate their functional roles in NAFLD.

## Materials and methods

### The high-cholesterol diet-fed adult zebrafish model

Forty wild-types AB-line adult zebrafish (3 months postfertilization) were included in an experiment. The water of the tank was maintained at 28 °C. All 40 zebrafish were fed a high-cholesterol diet (1.5 g per day) for 8 weeks. A high-cholesterol diet was prepared according to the study of Ji Ma et al.^14^ Gemma micro (ZF 300, Skretting, United States) was used as basic food. After 8 weeks, 36 zebrafish fed high-cholesterol diets were anesthetized with 0.2% 10 g of ethyl3-aminobenzoate methanesulfonate (Sigma, #E10521) for 3 to 5 min and euthanized. Four zebrafish died during the experimental period. The workflow diagram of zebrafish feeding and tissue processing is described and illustrated in Fig. 1 using Microsoft PowerPoint 2013 and Adobe draw. This study was approved by the Gyeongsang National University Institutional Animal Care and Use Committee (GNU-201012-E0074). In addition, all experiments involving live animals were performed in accordance with relevant guidelines and regulations and were reported as described by the recommendations in the ARRIVE guidelines.

### Measurement of fatty changes in the liver using oil red O staining

**Figure 6:**
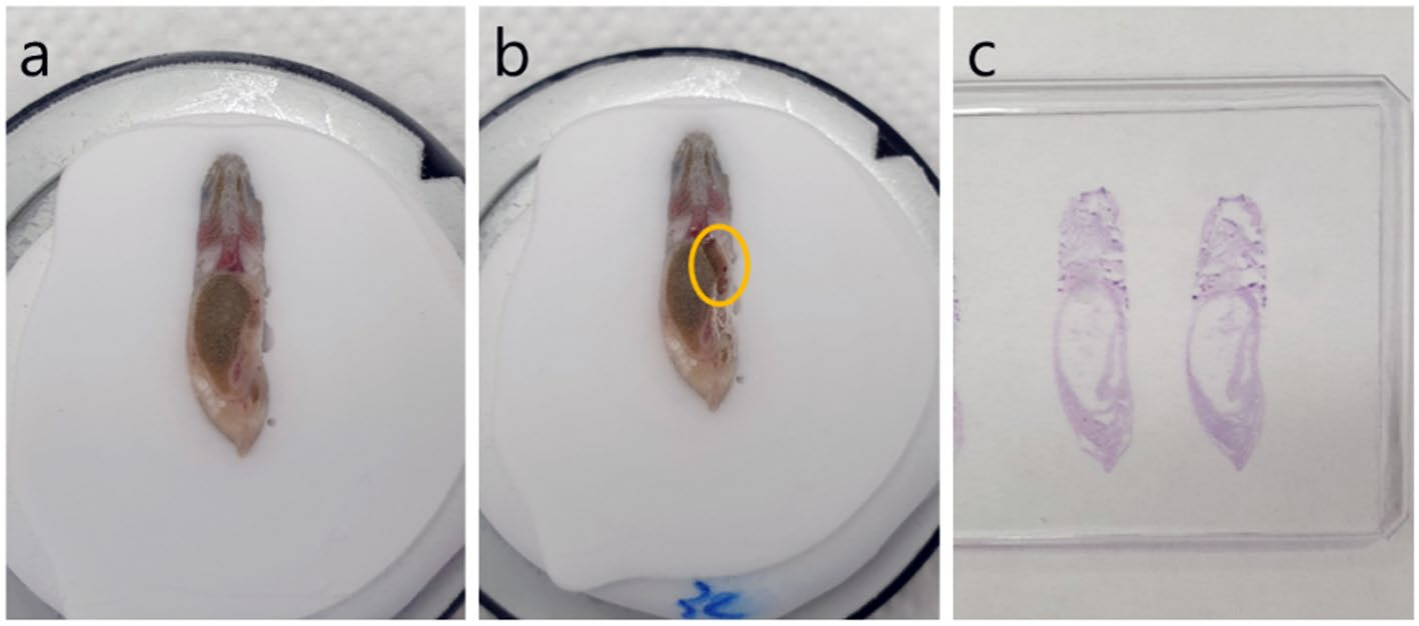
Frozen sections of each zebrafish with longitudinal sections were used for fat staining. **(a)** When the liver (circled) began to appear on the cut surface **(b)** of the frozen section, it was cut into serial sections with a thickness of 4 µm **(c)**.

We used frozen sections of each zebrafish (Fig. 6a) and cut them into longitudinal sections for fat staining. When the liver that surrounds the stomach, the largest organ in the zebrafish, began to appear in the cut surface (Fig. 6b) of the frozen section, it was cut into serial sections with a thickness of 4 µm (Fig. 6c), and oil red O staining was performed on the best section. We obtained six or more frozen liver sections from each zebrafish and performed oil red O staining according to the modified method of our previous study^15^. Sections were cut at a thickness of 4 µm. In addition, 80% propylene glycol was added for 2 min, and oil red O staining was performed for 35 min. After washing the dyed slides twice with distilled water, further washing was carried out microscopically to prevent overstaining. Otherwise, the method was the same as in our previous study^15^. The proportion of fatty changes on a slide with oil red O staining was evaluated by two pathologists through a blinded method. The highest proportional value among the six sections of each zebrafish was the representative score.

### RNA extraction and Next-generation sequencing analysis

Each piece of liver tissue was obtained from the remaining frozen specimen using a scalpel. Then, those tissues were immediately crushed in 10 µl of quiazole and stored at -70 °C. Liver specimens of zebrafish numbers 4, 15, 22, 25, 32, 34, and 36 with high fatty liver scores based on oil red O staining were pooled into a “high” group. Zebrafish numbers 1, 5, 11, 13, 14, 20, and 29 were pooled into a “low” group. For RNA-sequencing, we extracted RNA from two pooled groups. The method of RNA extraction from the tissue is described in Supplementary data 4. We performed library preparation and validation quality check before the Next-generation sequencing. Rebo-depletion was performed by Ribo-zero H/M/R Gold, during the library preparation. Total RNA integrity was checked using an Agilent Technologies 2100 Bioanalyzer with an RNA Integrity Number (RIN) value greater than or equal to 7. To verify the size of PCR enriched fragments, template size distribution was checked running on an Agilent Technologies 2100 Bioanalyzer using a DNA 1000 chip. Transcriptome sequencing was performed using an Illumina platform. The raw data of sequencing were extracted as fragments per kilobase of exon per million fragments mapped (FPKM) across each sample. The statistical significance in the fold change of transcript expression profile was determined by paired t-tests (Macrogen, https://www.macrogen.co.kr). Data of the transcriptome sequencing and gene ontology analyses are summarized in Supplementary data 5 and 2, respectively.

## Supplementary Information

Dataset S1.

Supplementary Information.
