# Supplementary material for: Long noncoding RNAs associated with nonalcoholic fatty liver disease in a high cholesterol diet adult zebrafish model
Source: Sci Rep. 2021 Nov 26;11:23005. doi: 10.1038/s41598-021-02455-0 (PMC8626429; doi:10.1038/s41598-021-02455-0)
Supplement: Supplementary file 2 — Supplementary Information. [file 41598_2021_2455_MOESM2_ESM.docx]

Supplementary information

**Long Noncoding RNAs Associated with Nonalcoholic Fatty Liver Disease in a High Cholesterol Diet Adult Zebrafish Model**

Running title: LncRNAs associated with NAFLD

Hyo Jung An^1,2,3^, Yoon Jung Lee^3^, Chong Pyo Choe^4,5^, Hyun-Kyung Cho^6,7^, and Dae Hyun Song^1,2,3*^

^1^ Department of pathology, Gyeongsang National University Changwon Hospital, Changwon, Republic of Korea;

^2^ Department of pathology, Gyeongsang National University School of Medicine, Jinju, Republic of Korea;

^3^ Department of pathology, College of Medicine, Institute of Health Science, Gyeongsang National University, Jinju, Republic of Korea;

^4^ Division of Life Science, Gyeongsang National University, Jinju 52828, Republic of Korea.

^5^ Division of Applied Life Science, Plant Molecular Biology and Biotechnology Research Center, Gyeongsang National University, Jinju, Republic of Korea.

^6^ Department of Ophthalmology, Gyeongsang National University Changwon Hospital, Gyeongsang National University, School of Medicine, Changwon, Republic of Korea;

^7^ Institute of Health Sciences, School of Medicine, Gyeongsang National University, Jinju, Republic of Korea.

**Corresponding author**

Dae Hyun Song, M.D.

Department of Pathology, Gyeongsang National University School of Medicine, 15 Jinju-daero 816 Beon-gil, Jinju 660-751, Korea

Tel: +82-55-214-3150

Fax: +82-55-214-3174

E-mail: [golgy@hanmail.net](mailto:golgy@hanmail.net)

Supplementary data 1.

The heatmap using the Z-score of the log 2 based Top 8 differentially expressed protein- coding genes with normalized value by Morpheus Software (<https://software.broadinstitute.org/morpheus/>)


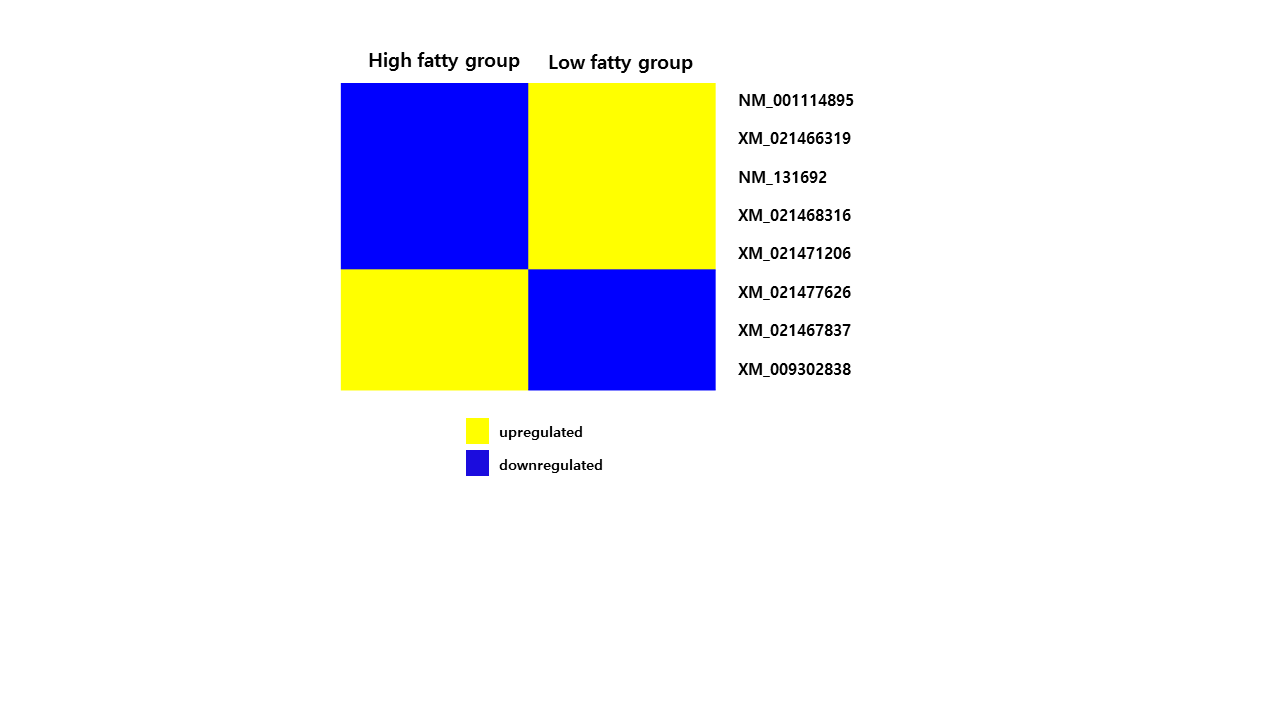


Supplementary data 2.

The Gene ontology (GO) term analyses of the regulated genes associated with NAFLD in zebrafish.


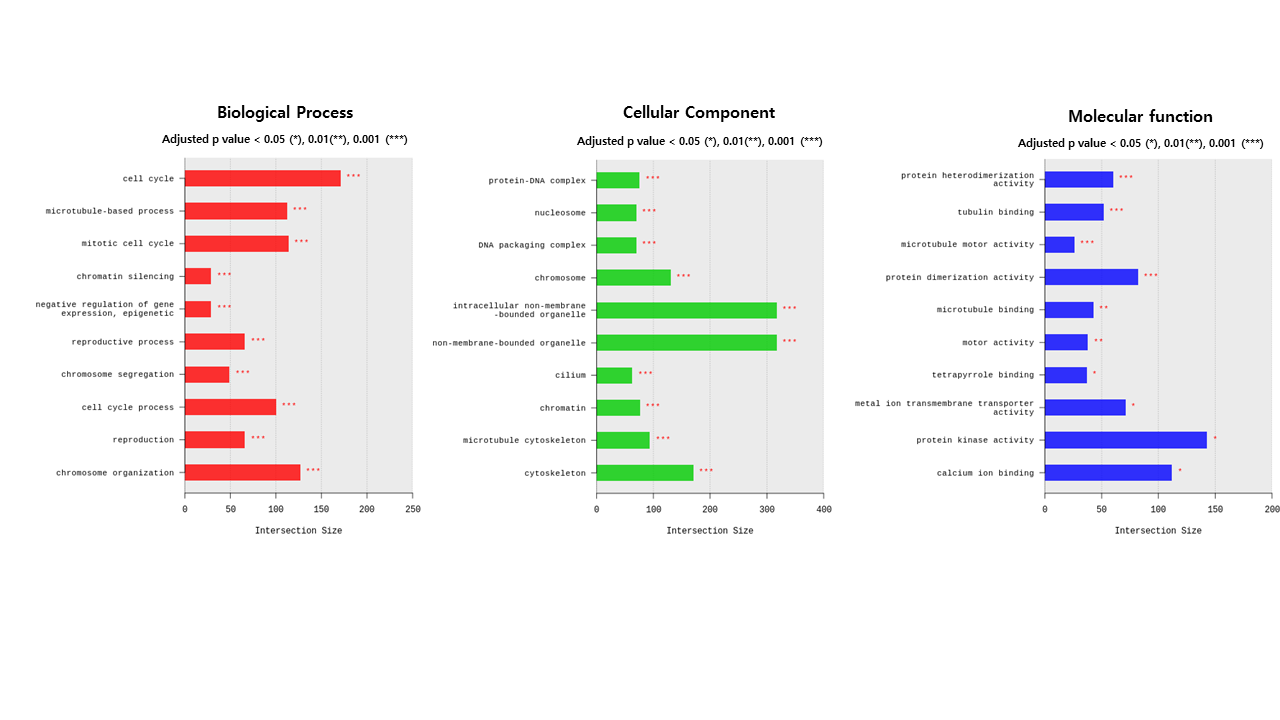


Supplementary data 3.

8 conserved lncRNAs with FASTA links.

1. NONHSAT149958 : http://www.noncode.org/show_rna.php?id=NONHSAT149958&version=1&utd=1#
2. ENST00000475761: https://asia.ensembl.org/Homo_sapiens/Transcript/Exons?db=core;g=ENSG00000242559;r=X:41683168-41683462;t=ENST00000475761
3. NONHSAT180755:

http://www.noncode.org/show_rna.php?id=NONHSAT180755&version=1&utd=1#

1. NONHSAT174445:

http://www.noncode.org/show_rna.php?id=NONHSAT174445&version=1&utd=1#

1. NONHSAT205239:

http://www.noncode.org/show_rna.php?id=NONHSAT205239&version=1&utd=1#

1. ENST00000415679: https://asia.ensembl.org/Homo_sapiens/Transcript/Exons?db=core;g=ENSG00000237409;r=1:160020300-160020727;t=ENST00000415679
2. ENST00000426250:

https://asia.ensembl.org/Homo_sapiens/Transcript/Exons?db=core;g=ENSG00000236333;r=12:72253538-72274907;t=ENST00000426250

1. NONHSAT030573: http://www.noncode.org/show_rna.php?id=NONHSAT030573&version=2&utd=1#

Supplementary data 4.

Trizol Cell RNA Extraction protocol

**Material**

- QIAzol® Lysis Reagent (Cat. No. 79306), Chloroform, Isopropanol(stored at-20°C),

70% ethanol(stored at -20°C), RNase-free water

**Method**

Homogenizing Samples

1. Add 750ul Trizol directly to the maximum of 1x10^7^ cells.

2. Pipette this mixture tenderly.

3. Incubate for 5 minutes at room temperature for lysis.

**Phase separation & RNA precipitation**

1. Add 150ul of chloroform per 750 ul of Trizol used for homogenization.

2. Shake the tube vigorously by vortex for 15 seconds.

3. Incubate for 2–3 minutes at room temperature.

4. Centrifuge the sample at 12,000 × g for 15 minutes at 4°C.

5. Transfer the upper aqueous phase to a new tube.

6. Add an equal volume of 100% isopropanol to the aqueous phase. Mix by inverting.

7. Incubate at room temperature for 10 minutes.

8. Centrifuge at 12,000 × *g* for 10 minutes at 4°C.

**RNA wash & Resuspension**

1. Remove the supernatant from the tube, leaving only the RNA pellet.

2. Wash the pellet, with 700 ul of 70% ethanol. Mix by inverting.

3. Centrifuge the tube at 12000 × *g* for 5 minutes at 4°C. Discard the supernatants.

4. Spin down at 12000 × *g* for 1 minute at 4°C. Remove the supernatants completely, and briefly air-dry the RNA pellets.

5. Resuspend the homogenized samples using 50 ul of RNase-free water.

Supplementary data 5.

| Species | Danio rerio |
| --- | --- |
| Reference | GRCz11 |
| Annotation | NCBI_106 |
| Library Kit | TruSeq Stranded Total RNA LT Sample Prep Kit (Gold) |
| Library protocol | TruSeq Stranded Total RNA Sample Prep Guide, Part#15031048 Rev. E |
| Type of Sequencer | Illumina platform |
